# Simultaneous Determination of Six Immunosuppressants in Human Whole Blood by HPLC-MS/MS Using a Modified QuEChERS Method

**DOI:** 10.3390/molecules27134087

**Published:** 2022-06-25

**Authors:** Min Zheng, Jianshi Song, Hua Xue, Hui Li, Kaoqi Lian

**Affiliations:** 1 School of Public Health, Hebei Medical University, Shijiazhuang 050017, China; zhengmin2163@163.com; 2 Jinan Center for Disease Control and Prevention, Jinan 250000, China; 3 The School of Basic Medicine, Hebei Medical University, Shijiazhuang 050017, China; jianshisong20@163.com; 4 Chemistry Teaching Group and Fundamental Medical Department, Shijiazhuang 050599, China; xuejingtai@163.com; 5 Hebei Institute for Drug and Medical Device Control, Shijiazhuang 050299, China; 6 Hebei Key Laboratory of Environment and Human Health, Shijiazhuang 050017, China

**Keywords:** QuEChERS, immunosuppressants, whole blood, HPLC-MS/MS

## Abstract

A high-performance liquid chromatography-tandem mass spectrometry method was established for the simultaneous determination of mycophenolic acid, mycophenolate mofetil, tacrolimus, rapamycin, everolimus and pimecrolimus in human whole blood by optimizing the QuEChERS (Quick, Easy, Cheap, Effective, Rugged, and Safe) preparation method. Whole blood was extracted into ethyl acetate, salted out with anhydrous magnesium sulfate, and purified with ethylenediamine-N-propyl silane adsorbent. The supernatant was evaporated under nitrogen until dry and finally reconstituted in methanol. Chromatographic separation was performed on an Agilent Poroshell 120 EC-C18 column in methanol (mobile phase A)-water (optimized for 0.1% acetic acid and 10 mM ammonium acetate, mobile phase B) at a 0.3 mL·min^−1^ flow rate. Electrospray ionization and positive ion multiple reaction monitoring were used for detection. The time for of analysis was 13 min. The calibration curves range of tacrolimus, rapamycin, everolimus and pimecrolimus were in the range of 1–100 ng·mL^−1^, mycophenolate mofetil in the range of 0.1–10 ng·mL^−1^ and mycophenolic acid at 10–1000 ng·mL^−1^. All correlation coefficients were >0.993. The coefficients of variation (CV, %) for inter-day and intra-day precision were less than 10%, while the spiked recoveries were in the range of 92.1% to 116%. Our method was rapid, sensitive, specific, and reproducible for the simultaneous determination of six immunosuppressants in human whole blood. Importantly, our approach can be used to monitor drug concentrations in the blood to facilitate disease treatment.

## 1. Introduction

Allorejection is a significant issue during allotransplantation procedures. Many immunosuppressive drugs can be used to suppress a recipient’s immune response by inhibiting abnormal immune responses in the body [1]. Based on functionality, immunosuppressive drugs are divided into four categories. The first category is represented by adrenocorticotropic hormones and includes prednisone and methylprednisone. The second category area is characterized by tacrolimus (FK-506) and other cytokine synthesis inhibitors. The third group includes immunosuppressants such as rapamycin (RAPA) and mycophenolate esters, which inhibit relevant signaling pathways via synergistic effects when combined with second-generation drugs. The fourth category includes monoclonal anti-lymphocyte antibodies. FK-506, RAPA, everolimus (EVER) and mycophenolate mofetil (MMF) are commonly used in clinical practice [2,3]. The widespread use of immunosuppressive drugs has not only improved the control of allogeneic rejection reactions but also greatly enhanced transplantation rates, thereby reinforcing their importance in the treatment of autoimmune diseases and diseases caused by allergic reactions [4,5]. However, significant adverse effects exist [6] including metabolic disorders, nephrotoxicity, and hyperlipidemia [7,8,9]. Therefore, the use of immunosuppressive drugs at adequate concentration is essential to facilitate optimal blood levels in patients for accurate drug use [10].

Several analytical methods have been developed [10], including chemiluminescence microparticle immunoassay [11], enzyme-multiplied immunoassay [12], microparticle enzyme immunoassay [13], enzyme-linked immune absorbent assay [14,15,16], high-performance liquid chromatography (HPLC), and liquid chromatography-tandem mass spectrometry (LC-MS/MS) [1,3,17]. Immunoassays have several drawbacks despite simple operating methods and specialized kit formats. Immunoassays do not identify analytes and their metabolites. In addition to high detection values, insufficient limits of quantitation (LOQ) and poor specificity are a cause for concern. Further, some drugs are extensively metabolized and cross-react with other metabolites, thereby affecting the results. The LC-MS/MS method is specific, sensitive, and reliable, and may eventually replace immunoassays as the primary monitoring method for the monitoring of circulatory immunosuppressants. As immunosuppressants are typically used in multiple drug combinations, rapid and effective LC-MS/MS techniques are required to simultaneously analyze various immunosuppressants in the blood. For example, FK-506 is often treated with MMF in kidney transplantation. Based on individual differences, other treatment regimens include FK-506/EVR/prednisolone (Perd), FK-506/mycophenolic acld (MPA)/Perd triple regimens, and FK-506/EVR double regimens [18,19,20,21].

In this study, a quick, easy, cheap, rugged, effective and safe (QuEChERS) method was used for sample preparation. Generated by Anastassiades and Lehotay [22], this method is based on solid-phase extraction and matrix-solid phase dispersion techniques [23,24,25]. The QuEChERS method is simple and rapid, requires less solvent for the extraction process, and is associated with less environmental pollution. The procedure was originally used to analyze pesticide residues in succulent fruits and vegetables [26,27]; however, technical improvements have led to its widespread use in the analysis of drug residues, metabolites, and compounds in blood. The modified method has three basic steps: (1) extraction of a homogeneous sample in organic solvent; (2) addition of the extracted sample to inorganic salts and separation of the organic layer; and (3) addition of sorbent to purify specific analytes [28]. When compared with conventional extraction methods, the modified QuEChERS method is simple and cheap, with a short processing time, low solvent consumption, and a high pigment purification rate.

Here, we combined the HPLC-MS/MS technique with the modified QuEChERS method for the extraction and purification of immunosuppressants from whole blood samples. Further, we developed a quantitative method to analyze the concentrations of MPA, MMF, FK-506, RAPA, EVER and pimecrolimus (PIM) in human whole blood. The method yielded a good limit of detection (LOD), LOQ, linear range, precision, accuracy, and matrix effects (ME). Thus, the approach provides a reference method for the detection of immunosuppressant concentrations and provides guidance for the clinical use of drugs.

## 2. Results

### Validation of the Analytical Method

**Figure 1:**
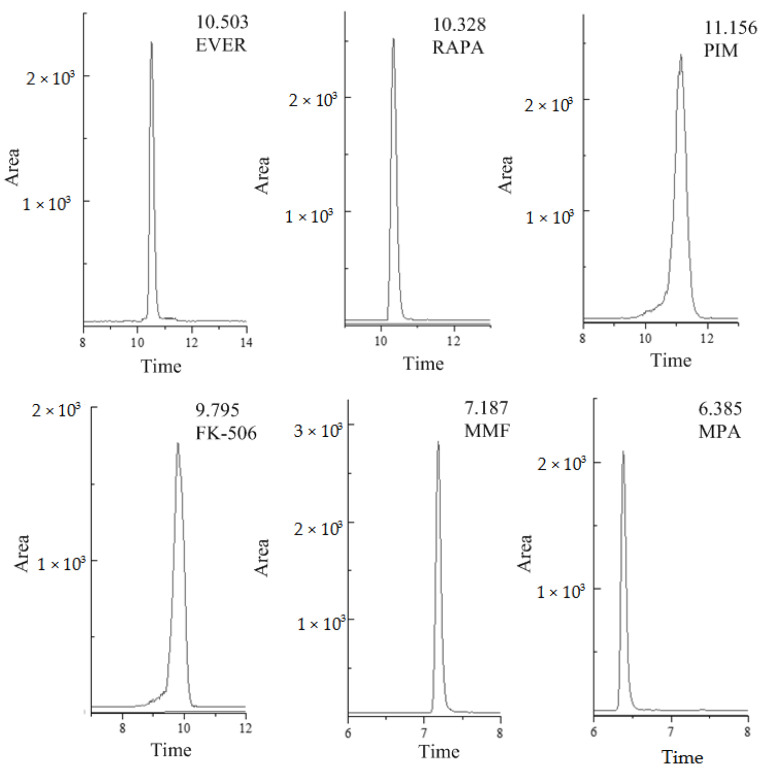
Typical selective ion chromatograms of blank plasma spiked reference compounds (FK-506 and PIM at 100 ng·mL^−1^, RAPA and EVER at 500 ng·mL^−1^, MMF at 10 ng·mL^−1^ and MPA at 1000 ng·mL^−1^), (MPA = Mycophenolic acid, MMF = Mycophenolate Mofetil, FK-506 = Tacrolimus, RAPA = Rapamycin, EVER = Everolimus, PIM = Pimecrolimus).

The HPLC-MS/MS method used in this research for the determination of six different compounds in human whole blood was fully validated. The selectivity testing allowed us to verify that no peaks from endogenous compounds during retention time correspond to each analyte and the interferences were less than 20% of LOQ signals. For the blank sample, there was no obvious interference peak in the enrichment detection of the analyte in this experiment. The selective ion chromatograms of human whole blood spiked with analytes are presented in Figure 1 (Agilent Poroshell 120 EC-C_18_ column). There was no obvious interference near the selective ion chromatogram. The ME of MMF and FK-506 was <80% (78.95% for MMF in QC samples of medium concentration, and 78.97% for FK-506 in high-concentration QC samples). RAPA and EVER showed significant matrix inhibition effects in medium and high concentration QC samples, with a substantial ME of approximately 50%, so the ME was not ignorable (Table 1). All matrix-matched calibration curves showed good linearity (r^2^ > 0.993) for all analytes (Table S1). The coefficients of variation (CV, %) for inter-day and intra-day precision were less than 10%, while the spiked recoveries were in the range of 92.1% to 116%, depending on the analyte. The method was sensitive, with LOQs in the range of 0.06–7.60 ng·mL^−1^, whereas LODs were in the range of 0.02–2.30 ng·mL^−1^. The results are shown in Table 2.

## 3. Discussion

### 3.1. Column and Mobile Phase Selection

**Figure 2:**
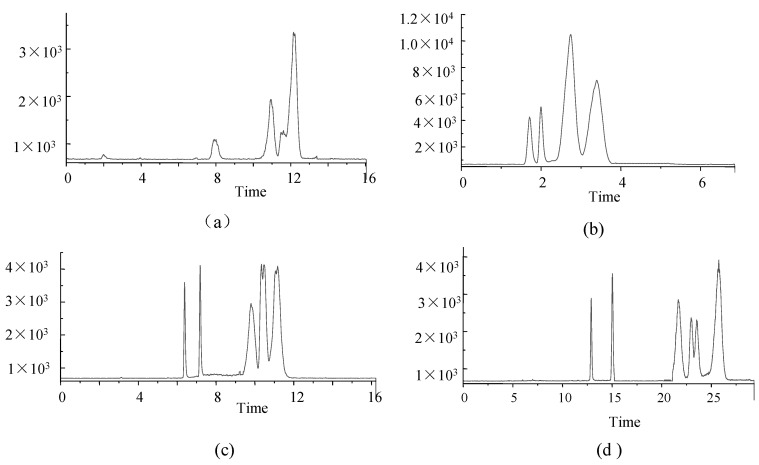
TIC of 6 analytes under four columns (**a**) Phenomenex Luna Omega 3 µm PS C18 100 Å; (**b**) Agilent Eclipse Plus C18; (**c**) Agilent Poroshell 120 EC-C18; (**d**) Agilent ZORBOX SB-C18. (FK-506 and PIM at 100 ng·mL^−1^, RAPA and EVER at 500 ng·mL^−1^, MMF at 10 ng·mL^−1^ and MPA at 1000 ng·mL^−1^).

The separation performances of four chromatographic columns were also assessed in this study. (1) Phenomenex Luna Omega 3 µm PS C_18_ 100 Å (2.1 mm × 150 mm); (2) Agilent Eclipse Plus C_18_ (3.0 × 100 mm, 1.8 μm); (3) Agilent Poroshell 120 EC-C_18_ (3.0 × 50 mm, 2.7 μm), and (4) Agilent ZORBOX SB-C_18_ (4.6 × 150 mm, 5 μm). Columns were used to compare the concentrations of target analytes for HPLC-MS/MS analysis (Figure 2).

The Phenomenex Luna Omega 3 µm PS C_18_ 100 Å column showed poor separation performance, low response values, and poor peak shapes (Figure 2a), probably due to small differences in the polarity of analytes.

The performance of the Agilent Eclipse Plus C_18_ column was poor, even though peak times were short and concentrated, and peak shapes were good (Figure 2b).

The Agilent Poroshell 120 EC-C_18_ column exhibited better retention of each compound, resolution and peak shape (Figure 2c) despite the similarity to the Agilent Poroshell 120 EC-C_18_ column in terms of separation and peak shape.

The Agilent ZORBOX SB-C_18_ column was longer, and analytes were slow to peak, and the analytical times were longer (Figure 2d).

Therefore, the Agilent Poroshell 120 EC-C_18_ column (Figure 2c) was the preferred analytical column in this study.

To determine the optimal organic phase, we evaluated MeOH and ACN and showed that analyte response values were higher when MeOH was used as the organic phase. Moreover, the peak shape of the target was effectively improved when ammonium acetate was added to the aqueous phase. In addition, the pH of the mobile phase also affected the peak shape and analyte response. Studies were conducted with water (plus 10 mM ammonium acetate)-MeOH, 0.1% acetic acid water (plus 10 mM ammonium acetate)-MeOH, 0.1% formic acid water (plus 10 mM ammonium acetate)-MeOH, 0.2% formic acid water (plus 10 mM ammonium acetate)-MeOH, and 0.5% formic acid water (plus 10 mM ammonium acetate)-MeOH. The optimal mobile phase solution was 0.1% acetic acid water (plus 10 mM ammonium acetate)-MeOH.

### 3.2. Selecting and Optimizing Preprocessing Conditions

#### 3.2.1. Optimizing Extraction Conditions

**Figure 3:**
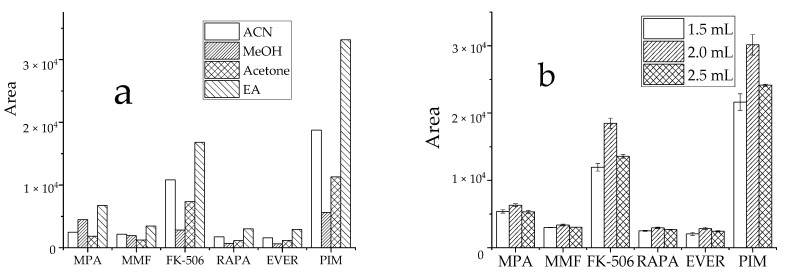
Effect of extractant on the recovery peak area of analyte, (**a**): types of extractant; (**b**): dosage of EA.

The efficiency of commonly used extraction solvents, such as ACN, MeOH, acetone, and EA was assessed by comparing the peak areas of target analytes in samples. For analysis, 1.5 mL organic extraction solvent (described earlier) was added to target analytes at similar concentrations. The precipitation of organic solvents is to reduce the dielectric constant of water, leading to dehydration, mutual aggregation, and biomolecule precipitation within the surface water layer. Due to the complex matrix in whole blood and associated influencing factors, EA displayed a more effective extraction efficiency, with good peak shape and less interference (Figure 3a). To improve the extraction efficiency and reduce the consumption of organic solvents, the extraction solvent levels were optimized. When 1.5 mL, 2 mL, and 2.5 mL EA (Figure 3b) were compared, the 2 mL EA displayed the highest detection peak area and had the best performance for target analytes. Therefore, 2 mL EA was used as the extraction solvent volume.

#### 3.2.2. Optimization of Salinization Conditions

**Figure 4:**
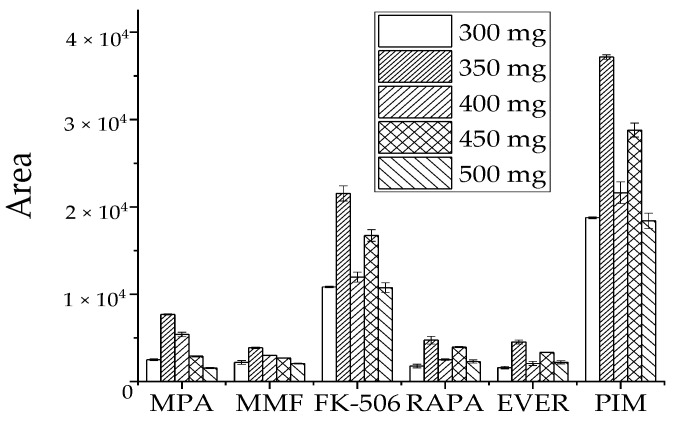
Effect of MgSO4 dosage on recovery peak area of target analyte.

High water levels in blood samples may affect instrument response, reduce recovery rates, and cause unnecessary losses in the column and mass spectrometry. We selected the dose of MgSO_4_ as the water removal agent by comparing the detection peak area of the target in the sample. Similar concentrations of the target analyte were treated with 300 mg, 350 mg, 400 mg, 450 mg, and 500 mg of MgSO_4_ (Figure 4). The peak target analyte area was the largest at 350 mg MgSO_4_, while the response value of each target analyte decreased when its dose was more or less than 350 mg. Therefore, 350 mg MgSO_4_ was selected as a dehydration reagent.

#### 3.2.3. Optimization of Purification Conditions

**Figure 5:**
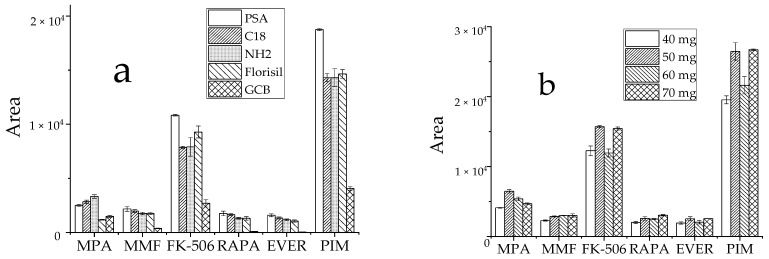
Effect of purifying agent on the recovery peak area of analyte, (**a**): types of purifiers; (**b**): dosage of PSA.

Commonly used adsorbents include PSA, GCB, Florisil, C18 and NH_2_ [29,30,31]. Typically, whole blood samples are complex and contain not only water, proteins, fats, phospholipids, and other substances, but also red and white blood cells and other blood constituents, all of which impact drug detection. Thus, these five adsorbents were selected to assess the effects of purification. Approximately 60 mg of each adsorbent was added at the same target analyte concentration, and the peak area of the detected target analyte in the sample was used to evaluate the purification effect. Except for MPA, all five target analytes were optimally purified by PSA (Figure 5a). Since the amount of purifying agent also had a large effect on the outcome, PSA concentrations of 40 mg, 50 mg, 60 mg, and 70 mg were tested to select the optimal amount at similar concentrations of the target analytes. The results showed that the target analyte responses differed little when the concentrations of the purifying agent were 50 mg and 70 mg, and the MPA response value reached the highest at 50 mg (Figure 5b). Based on low reagent consumption and cost savings, 50 mg PSA was selected as the ideal purification adsorbent.

#### 3.2.4. Comparative Analysis of QuEChERS and Solid-Phase Supported Liquid-Liquid Extraction (SLE)

To extract target analytes from whole blood samples and reduce matrix effects, two preparation methods, SLE and QuEChERS, were tested at the same target analyte concentration. Pretreatment efficiency was compared using the detected peak area magnitude. The optimal conditions for SLE were based on equal volumes of sample diluent pure water, eluent and EA, and 1.5 mL EA was added in three aliquots of 500 μL each. The optimal conditions for QuEChERS were characterized by 2.0 mL EA of extractant, 350 mg MgSO_4_ as the water removal agent, and 50 mg PSA as a purifying agent.

**Figure 6:**
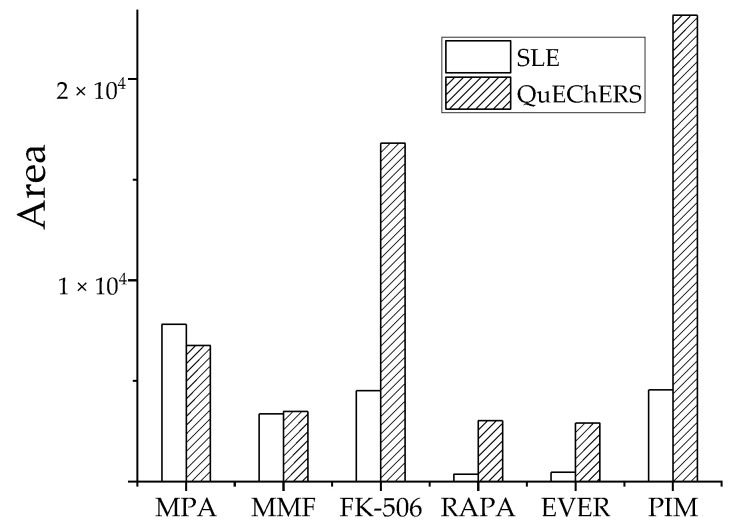
Effect of two pretreatment methods on recovery peak area of analyte.

Three parallel samples from medium-level QC samples were tested and each sample was measured in triplicate. The peak areas of analytes in each treatment group are shown (Figure 6). Based on target analyte response, the QuEChERS method was significantly better than SLE for FK-506, RAPA, EVER, and PIM. The pretreatment effect of MMF was comparable and the extraction effect of QuEChERS was slightly lower than SLE for MPA. Therefore, the optimized QuEChERS method was selected for sample preparation.

### 3.3. Comparisons with Other Methods

Our method was compared with other methods reported in the literature in terms of LOD and recovery (Table 3). The isolation and enrichment methods for immunosuppressants reported previously show limitations. As immunosuppressants exhibit non-linear binding to erythrocytes, whole blood samples are mostly used for analyses. Thus, when compared with whole blood samples, plasma or serum samples are easier to test but yield inaccurate results [32]. The solid-phase extraction (SPE) and organic solvent extraction preparation methods for precipitated proteins (PP) reported previously have lower detection limits or lower recoveries than the pretreatment methods in this study for MMF, FK-506, RAPA, PIM, MPA and EVER [33,34,35,36]. Compared with whole blood, cerebrospinal fluid is more difficult to collect in experiments, so our matrix used human whole blood [37]. The current method is characterized by increased sensitivity and recovery advantage compared with the previously published methods. The modified QuEChERS method is economical and effective, with good recovery, precision, and accuracy.

## 4. Materials and Methods

### 4.1. Quantitative Analysis by UHPLC-MS/MS

#### 4.1.1. Reagents and Chemicals

Standards: MMF, RAPA, EVER and PIM were purchased from Toronto Research Chemicals (North York, ON, Canada); MPA and FK-506 were purchased from the National Institute for Food and Drug Control (Beijing, China). Chromatographically pure acetonitrile (ACN), methanol (MeOH), formic acid and acetic acid were purchased from Dima Technology (Beijing, China); chromatographically pure acetone and ethyl acetate (EA) were purchased from Safran Technology (Tianjin, China); octadecyl-bonded silica gel (C18), *N*-propyl ethylenediamine adsorbent (PSA), Florisil, amino-bonded silica gel (NH2), graphitized carbon (GCB) were purchased from Agela Technologies (Tianjin, China); anhydrous magnesium sulfate (MgSO_4_) was purchased from Tianjin Damao Chemical Reagent Factory (Tianjin, China).

#### 4.1.2. Preparation of the Standard Stock Solutions and Working Solutions

The refined weighing MPA, MMF, FK-506, RAPA, EVER and PIM standard drugs were dissolved in MeOH. A stock solution with a mass concentration of 500 μg·mL^−1^ for each drug was stored in a −20 °C refrigerator. The standard curve working solution and the quality control working solution were diluted proportionally with MeOH to mix the 6 drugs. The concentrations of standard curve solution of FK-506, RAPA, EVER and PIM ranged from 1 ng·mL^−1^ to 100 ng·mL^−1^ (1, 5, 20, 50, 75 and 100 ng·mL^−1^ ); MPA ranged from 10 ng·mL^−1^ to 1000 ng·mL^−1^(10, 50, 200, 500, 750 and 1000 ng·mL^−1^); MMF ranged from 0.1 to 10 ng·mL^−1^ (0.1, 0.5, 2, 5, 7.5 and 10.0 ng·mL^−1^).

Low-, medium-, and high-concentration quality control (QC) samples were prepared by adding different volumes (10 μL aliquots) of mixed standard solutions to 500 μL of blank whole blood samples. Low, medium, and high FK-506, RAPA, EVER and PIM concentrations were 2, 50, and 80 ng·mL^−1^, respectively. Similarly, the three MPA concentrations were 20, 500, and 800 ng·mL^−1^, respectively, and the three MMF levels were 0.2, 5, and 8 ng·mL^−1^, respectively.

#### 4.1.3. Sample Preparation

Peripheral venous blood from volunteers was collected into disposable anticoagulation blood collection tubes. The whole blood samples were pretreated with the optimized QuEChERS method and stored at −20 °C. For analysis, blood was thawed at room temperature and vortexed. Then, 500 μL blood and 2.0 mL EA were added to a centrifuge tube and vortexed for 30 s, followed by the addition of 350 mg MgSO_4_ and 50 mg PSA adsorbent for purification. The tube was vortexed for 60 s and centrifuged at 12,000 rpm for 10 min. The supernatant was removed, dried under nitrogen, and re-dissolved in MeOH.

#### 4.1.4. LC-MS/MS Analysis

Electrospray ionization (ESI), Positive ion mode, Multiple reaction monitoring (MRM), Drying gas (Drying Gas) flow rate: 11 L·min^−1^, Drying gas temperature: 300 °C, Capillary voltage: 4000 V. The analytes, the monitored ions, the retention time, collision energy and fragmentation voltage are shown in Table 4.

The chromatographic column was an Agilent Poroshell 120 EC-C_18_ column (3.0 × 50 mm, 2.7 μm) from Agilent ( CA, USA); mobile phase A was water containing 0.1% acetic acid and 10 mM ammonium acetate, and mobile phase B was MeOH. The gradient elution procedure was as follows: 0~1.0 min, 5% B; 1.0~1.5 min, 5~45% B; 1.5~3.0 min, 45~90% B; 3.0~10.0 min, 90% B; 10~10.1 min, 90~5% B; 10.1~13 min, 5% B; the flow rate was 0.3 mL·min^−1^, the column temperature was 40 °C, and the injection volume was 5 μL.

### 4.2. Method Validation

The following parameters were established: linearity, selectivity, precision, recovery and ME. The selectivity was evaluated by analyzing different samples. Two blank whole blood samples were taken, one of which was spiked with the control mixture solution (FK-506 and PIM at 100 ng·mL^−1^, RAPA and EVER at 500 ng·mL^−1^, MMF at 10 ng·mL^−1^ and MPA at 1000 ng·mL^−1^). Both samples were processed and analyzed by HPLC-MS/MS.

Blank whole blood samples were added to the working solution of the control mixture and processed to generate a standard mixture of whole blood at the following final concentrations: FK-506, RAPA, EVER and PIM at 1, 5, 20, 50, 75, and 100 ng·mL^−1^; 0.1, 0.5, 2, 5, 7.5, and 10 ng·mL^−1^ for MMF, and 10, 50, 200, 500, 750, and 1000 ng·mL^−1^ for MPA. Linear regression analysis was performed with the horizontal coordinate represented by the mass concentration of each substance to be measured in whole blood (*X*), and the vertical coordinate indicated the peak area of the substance to be measured (*Y*). The LOD was calculated at three times the signal-to-noise ratio (S/N), and LOQs were calculated at ten times the S/N.

Blank whole blood samples were used to prepare QC low, medium, and high concentration samples, and processed. Three parallel samples were tested for each QC concentration and measurements continued for 3 days. Method precision and recovery were determined by comparing the added mass concentration with the measured mass concentration of samples.

An appropriate volume of blank human whole blood was processed to generate a blank whole blood matrix solution (the reconstitution solution was methanol). To this, a working solution of the control mixture of the six drugs was separately added to generate three mass concentration levels: low, medium, and high. The measured peak area (A) of the test substance was compared with the peak area (B) of the test substance obtained by direct injection of the corresponding mass concentration of the standard solution, and the ME (A/B × 100%) of this method was calculated. An ME between 80% and 120% indicated that substrate enhancement/inhibition was acceptable and negligible.

## 5. Conclusions

We simultaneously detected six immunosuppressive drugs in human whole blood using LC-MS/MS and a modified QuEChERS method. Compared with the conventional QuEChERS method, our method optimized and improved extractant conditions, salting and purifying agents. The validated method can be used for cost-effective immunosuppressant detection in whole blood with good precision, accuracy, and within-range linearity. The method is therefore ideal for the accurate detection of drug concentrations in patients with autoimmune disease, diseases caused by hypersensitivity reactions, and those undergoing transplantation.

## Figures and Tables

**Table 1:** Intra-day and inter-day precision and ME of analytes (*n* = 3).

Analyte	Spiked (ng·mL^−1^)	ME (%)	Precision (RSD %)		Accuracy (%)
			Inter-Day	Intra-Day	
MPA	20	101.99	3.52	1.13	107.79
	500	85.78	2.94	3.44	114.95
	800	103.19	6.73	2.31	110.75
MMF	0.2	112.69	6.44	2.78	109.56
	5	86.25	1.29	7.71	109.60
	8	78.95	3.76	1.33	106.42
FK-506	2	91.23	9.98	3.88	95.66
	50	80.51	0.55	1.36	109.09
	80	78.97	1.40	1.19	101.50
RAPA	2	94.08	4.31	5.49	109.43
	50	92.29	5.40	0.54	113.47
	80	47.81	3.05	4.94	113.09
EVER	2	106.65	14.92	4.59	92.24
	50	51.42	5.02	3.20	102.22
	80	50.26	2.07	1.61	102.44
PIM	2	91.17	7.02	2.98	85.07
	50	84.21	0.23	4.79	99.30
	80	85.20	0.86	1.01	92.07

**Table 2:** Validation parameters.

Analyte	Inter-Day (CV, %)	Intra-Day (CV, %)	LOD (ng·mL^−1^)	LOQ (ng·mL^−1^)	Recovery (%)
MPA	4.40 ± 2.0	2.29 ± 1.2	2.30	7.60	116 ± 3.6
MMF	3.83 ± 2.6	3.94 ± 3.3	0.02	0.07	109 ± 1.8
FK-506	3.98 ± 5.2	2.14 ± 1.5	0.03	0.09	102 ± 6.7
RAPA	4.25 ± 1.2	3.66 ± 2.7	0.05	0.20	112 ± 2.2
EVER	7.34 ± 6.7	3.13 ± 1.5	0.05	0.20	99.0 ± 5.8
PIM	2.70 ± 3.8	2.93 ± 1.9	0.02	0.06	92.1 ± 7.1

**Table 3:** Comparison of the developed method to the other approaches used in the extraction of immunosuppressors.

Method	Solvent	Sample	Analytes	Detection System	LOQ (ng·mL^−1^)	RSD (%)	Recovery (%)	Ref.
QuEChERS	-	Whole blood	MPA/MMF/FK-506/ RAPA/EVER/PIM	LC-MS/MS	7.60/0.07/0.09/0.20/0.20/0.06	0.23~14.92	85.07~114.95	this work
PP	Methanol	Plasma	FK-506	LC-MS/MS	0.1	-	107%	[32]
Online SPE	ZnSO4	Plasma	MPA/ FK-506/ RAPA/ EVER	LC-MS/MS	7.0/7.5/ 4.6/6.4	0.9~14.7	89.0~138.0	[33]
PP	ACN/ MTBE	Whole blood	FK-506/ RAPA/EVER	LC-MS/MS	0.5/0.5/ 0.5	1.87~11.2	78.6~85.9	[34]
Online SPE	MeOH- ZnSO4 (66:34)	Whole blood	FK-506/ RAPA/ EVER	LC-MS/MS	1.4/0.72/1.15	<5	92.8~95.9	[35]
PP	ZnSO4/ ACN	Whole blood	FK-506	LC-MS/MS	1.0	-	94	[36]
SPE	-	Brain	EVER	LC-MS	4.0	3~19	82~102	[37]

**Table 4:** The MS/MS fragment ions, fragmentor voltage, collision voltage and retention time of the 6 immunosuppressors.

Analyte	Precursor Ion (*m*/*z*)	Product Ion (*m*/*z*)	Fragmentor Voltage (V)	Collision Voltage (eV)	Retention Time (min)
MPA	338.3	207.1 *	140	20	6.4
	338.3	302.9	140	10	
MMF	434.2	114.2 *	145	28	7.2
	434.2	285.0	145	26	
FK-506	821.5	768.2 *	165	18	9.8
	821.5	786.2	165	14	
RAPA	931.5	864.4 *	155	15	10.3
	931.5	882.2	155	6	
EVER	975.5	908.3 *	165	10	10.5
	975.5	926.2	165	6	
PIM	827.4	774.2 *	170	20	11.2
	827.4	792.2	170	18	

* Quantitative ion.

## Data Availability

Data is contained within the article.

## Supplementary Materials

The following supporting information can be downloaded at: https://www.mdpi.com/article/10.3390/molecules27134087/s1, Table S1: Intra-day and inter-day precision and accuracy of analytes (*n* = 3); Table S2: Regression equations and limit of quantification of analytes.

## Sample Availability

Samples of the compounds (FK-506, PIM, RAPA, EVER, MMF, MPA) are available from the authors.
