# Supplementary material for: Simultaneous Determination of Six Immunosuppressants in Human Whole Blood by HPLC-MS/MS Using a Modified QuEChERS Method
Source: Molecules. 2022 Jun 25;27(13):4087. doi: 10.3390/molecules27134087 (PMC9268670; doi:10.3390/molecules27134087)
Supplement: Supplementary file 1 [file molecules-27-04087-s001.zip › molecules-1763853-supplementary.pdf]

molecules

*Supplemetary Material*

# Simultaneous Determination of Six Immunosuppressants in Human Whole Blood by HPLC-MS/MS Using a Modified QuEChERS Method

**Min Zheng [1,2], Jianshi Song [3], Hua Xue [4], Hui Li [5,*] and Kaoqi Lian [1,6,*]**

[1] School of Public Health, Hebei Medical University, Shijiazhuang 050017, China; zhengmin2163@163.com
[2] Jinan Center for Disease Control and Prevention, Jinan 250000, China
[3] The School of Basic Medicine, Hebei Medical University, Shijiazhuang 050017, China; jianshisong20@163.com
[4] Chemistry Teaching Group and Fundamental Medical Department, Shijiazhuang 050599, China; xuejingtai@163.com
[5] Hebei Institute for Drug and Medical Device Control, Shijiazhuang 050299, China
[6] Hebei Key Laboratory of Environment and Human Health, Shijiazhuang 050017, China
* Correspondence: lihui7171@163.com (H.L.); liankq@hebmu.edu.cn (K.L.); Tel.: +86-0311-69086009 (H.L.); +86-0311-86261043 (K.L.)

**Table S1.** Intra-day and inter-day precision and accuracy of analytes ($n$ = 3).

| Compounds | Spiked (ng·mL$^{-1}$) | ME (%) | Precision (RSD %) | | Recovery (%) |
|---|---|---|---|---|---|
| | | | Inter-day | Intra-day | |
| MPA | 20 | 101.99 | 3.52 | 1.13 | 107.79 |
| | 500 | 85.78 | 2.94 | 3.44 | 114.95 |
| | 800 | 103.19 | 6.73 | 2.31 | 110.75 |
| MMF | 0.2 | 112.69 | 6.44 | 2.78 | 109.56 |
| | 5 | 86.25 | 1.29 | 7.71 | 109.60 |
| | 8 | 78.95 | 3.76 | 1.33 | 106.42 |
| FK-506 | 2 | 91.23 | 9.98 | 3.88 | 95.66 |
| | 50 | 80.51 | 0.55 | 1.36 | 109.09 |
| | 80 | 78.97 | 1.40 | 1.19 | 101.50 |
| RAPA | 2 | 94.08 | 4.31 | 5.49 | 109.43 |
| | 50 | 92.29 | 5.40 | 0.54 | 113.47 |
| | 80 | 47.81 | 3.05 | 4.94 | 113.09 |
| EVER | 2 | 106.65 | 14.92 | 4.59 | 92.24 |
| | 50 | 51.42 | 5.02 | 3.20 | 102.22 |
| | 80 | 50.26 | 2.07 | 1.61 | 102.44 |
| PIM | 2 | 91.17 | 7.02 | 2.98 | 85.07 |
| | 50 | 84.21 | 0.23 | 4.79 | 99.30 |
| | 80 | 85.20 | 0.86 | 1.01 | 92.07 |

**Table S2.** Regression equations and limit of quantification of analytes.

| Compounds | Linear equation | Linear range (ng·mL$^{-1}$) | $R^2$ | LOD (ng·mL$^{-1}$) | LOQ (ng·mL$^{-1}$) |
|---|---|---|---|---|---|
| MPA | $Y = 2.2665x + 33.029$ | 10–1000 | 0.9935 | 2.30 | 7.60 |
| MMF | $Y = 470.01x + 19.641$ | 0.10–10.0 | 0.9992 | 0.02 | 0.07 |
| FK-506 | $Y = 307.77x - 193.5$ | 1.0–100 | 0.9984 | 0.03 | 0.09 |
| RAPA | $Y = 77.969x - 205.36$ | 1.0–100 | 0.9933 | 0.05 | 0.20 |
| EVER | $Y = 73.954x - 186.77$ | 1.0–100 | 0.9978 | 0.05 | 0.20 |
| PIM | $Y = 678.47x + 115.07$ | 1.0–100 | 0.9966 | 0.02 | 0.06 |
